# Physical Activity, Obesity, and Hypertension among Adults in a Rapidly Urbanised City

**DOI:** 10.1155/2021/9982562

**Published:** 2021-08-11

**Authors:** Qianqian Dun, Wanglin Xu, Maozhen Fu, Nengjian Wu, Justin B. Moore, Ting Yu, Xin Li, Yating Du, Biao Zhang, Qiaomai Wang, Yiting Duan, Ziqi Meng, Shuangshuang Tian, Yuliang Zou

**Affiliations:** ^1^School of Health Sciences, Wuhan University, Wuhan, Hubei 430071, China; ^2^Disease Control and Prevention Center of Pingshan District in Shenzhen City, Guangzhou 518100, China; ^3^Department of Implementation Science, Wake Forest School of Medicine, Medical Center Boulevard, Winston-Salem, NC 27157, USA; ^4^School of Urban Design, Wuhan University, Wuhan, Hubei 430072, China; ^5^School of Nursing, Weifang Medical University, Weifang, Shandong 261053, China

## Abstract

**Background:**

Few studies have explored the relationship between the level of physical activity and the occurrence or prevalence of obesity and hypertension among people residing in urbanised areas.

**Method:**

A cross-sectional study involving a sample of 1,001 adults was conducted. Descriptive statistics were used to describe sociodemographic variables, physical activity levels, body mass index (BMI), and prevalence of hypertension. Logistic regression models were adopted to investigate the relationship between these factors.

**Results:**

A total of 939 respondents who provided valid responses were included. Among them, 56.5% of the participants reported engaging in high levels of physical activity. However, 40.4% of the respondents were classified as overweight or obese, and 31.9% had diagnosed hypertension. After adjusting for sociodemographic factors, logistic regression analysis revealed that physical activity levels were negatively correlated with the prevalence of BMI (OR = 0.564, 95% CI: 0.352–0.905; OR = 0.583, 95% CI: 0.375–0.907) and hypertension (OR = 0.556, 95% CI: 0.348–0.888).

**Conclusions:**

Our study confirms recent evidence regarding the amount of physical activity that is associated with lower prevalence of obesity and hypertension in Pingshan District. Furthermore, different physical activities of various intensity levels had different effects on hypertension. Residents should be encouraged to engage in physical activities and maintain a healthy weight to improve their quality of life.

## 1. Introduction

Hypertension, also known as elevated blood pressure (BP), is a disease that occurs when blood vessels are constantly strained by high pressure [1]. Over 90% of patients with hypertension of unknown causes are diagnosed with primary hypertension. By contrast, 5%–10% of patients with hypertension suffer from other diseases, a condition called secondary hypertension. Hypertension is the most common cardiovascular disease (CVD) in China and a major public health problem worldwide [2]. According to the 2015 report on the status of “Nutrition and Chronic Diseases of Chinese Residents,” the prevalence of hypertension among adults in China was 25.2%, and the prevalence of this disease in urban areas (26.8%) was higher than that in rural areas (23.5%). Moreover, the prevalence was higher in men than in women, and it notably increased with age [3]. According to the Sixth National Census (2010), the number of patients with hypertension in China was estimated to be 270 million [4]. In 2013, the direct economic burden caused by hypertension accounted for 6.6% of the total health costs in the country [5].

With rapid urbanisation and ageing in China, the prevalence of hypertension is increasing, thereby imposing a serious economic and disease burden to society [6]. Numerous studies reported that high BP increases the risk of developing common chronic diseases, such as heart, brain, and kidney diseases, as well as the occurrence of cerebrovascular accidents and coronary heart disease; moreover, hypertension negatively affects the health of pregnant women and newborn children [7–10]. Perumareddi [11] demonstrated that hypertension is clinically associated with the risks of CVDs, three arteriosclerotic diseases, and other complications, such as congestive heart failure and cerebrovascular diseases. Ettehad et al. [12] argued that lowering BP can effectively prevent CVDs and death. Therefore, the occurrence and development of hypertension must be prevented and controlled to reduce the risk of CVDs and cerebrovascular diseases.

Hypertension is a disease caused by a combination of multiple factors. Gene-gene, gene-environment, and environment-environment interactions lead to different populations and individuals at different risk of developing hypertension [13]. Current studies proposed that the incidence of hypertension involves two main aspects: one is genetic, that is, individuals having different levels of genetic susceptibility, and the other is environmental [14, 15]. From a social ecological perspective [16], individual behaviour is strongly correlated with hypertension because physical activity (or the lack of it) and dietary intake determine if a person will develop obesity [17]. Furthermore, overweight and obesity are important risk factors affecting the incidence of hypertension [18]. You et al. [19] showed that even 10 min of vigorous physical activity every week can help reduce the prevalence of hypertension. Several studies abroad and in the country have examined the relationship between physical activity and hypertension [19–22].

With the development of society and the introduction of health policies, people pay more attention to health. At the same time, the urbanisation process began in the late 1970s and has dramatically increased its pace during the past two decades [23]. Rapid urbanisation has posed additional challenges to the population, who has to deal with the double threats of worsening/unhealthy diets due to increasing availability of unhealthy foods and decreasing physical activity due to exponential growth in car ownership and use. Therefore, the relationship between physical activity and prevalence of hypertension in this context must be examined.

In addition, the research area is Pingshan District, with an area of 168 km^2^, located in the northeast of Shenzhen City, Guangdong Province in southern China. This manufacturing-dominated region started urbanising in 2004, providing housing for hundreds of thousands of migrant workers. As a major residential area for migrant workers, the age distribution is positively skewed, and thus morbidity and mortality rates due to chronic diseases in this area are lower than those in other regions. However, the threat posed by unhealthy lifestyles remains high and may lead to higher prevalence of chronic diseases, such as obesity and hypertension. This is a young migrant population and a unique opportunity to study the impact of urbanisation on hypertension, PA, and obesity.

The aim of this study was to examine the relationship between physical activity and prevalence of obesity and hypertension in rapidly urbanising areas in the context of the “healthy city projects” of China. This study provides important insights into the burden of hypertension epidemic in relation to obesity due to the lack of physical activity in the population in urban areas. Moreover, this study offers guidance for formulating science-based strategies for hypertension prevention and control in Pingshan District. In future research, large-scale public health interventions on physical activity and obesity can be developed and tested.

## 2. Methods

### 2.1. Study Design and Sampling

A three-stage, stratified sampling strategy was employed. Six of the 16 communities in Pingshan District were randomly selected. From each sampled community, 150 families were selected. Finally, one member from each family between the age of 18 and 69 years was randomly selected via the Kish Grid sampling (one qualified subject from each family based on the principle of equal probability) [24]. A total of 1,001 adults were surveyed. Sixty-two questionnaires were excluded from the analysis due to missing data on key variables, resulting in a final sample of 939 respondents.

Various sociodemographic and physical information, such as gender, age, length of residency, education level, occupation, marital status, physical activity, and BMI (weight divided by the square of height), were collected through the questionnaires.

### 2.2. Assessment of Physical Activity

Self-reported data on physical activity during an entire week were collected via the long form International Physical Activity Questionnaire [25]. Data were collected from specific settings across several domains, including occupation, transportation, housework, and recreational activity, and reported in metabolic equivalents of task (MET) × minutes per week. MET is a physiological measure expressing the energy cost of physical activities, and it is defined as the ratio of metabolic rate (and therefore the rate of energy consumption) during a specific physical activity to a reference metabolic rate [26]. Walking MET was assigned with a ratio of 3.3, moderate-intensity activity with a ratio of 4.0, and high-intensity activity with a ratio of 8.0. Physical activity was calculated using the formula (MET − min) = MET × activity days per week (d) × daily activity time (min).

According to the total physical activity and the frequency of different types of physical activities within 1 week, physical activity was categorised into high, medium, and low [27]. High level satisfied guidelines for vigorous physical activity (≥three times per week, ≥1500 METS) or various physical activities (≥seven times per week, ≥3000 METS). Moderate level satisfied guidelines for vigorous physical activity (≥three times per week, ≥20 min per day), moderate physical activity/walking (≥five times per week, ≥30 min per day), or various physical activities (≥five times per week, ≥600 METS). Low level comprised two types: insufficiently active (some physical activity but less than the criteria for high or moderate level) or inactive (no physical activity reported).

### 2.3. BMI and BP Measurements

Objective anthropometric measures of body weight (kg) and height (m) were obtained from the study participants at home or through community visits. BMI was calculated as body weight in kg divided by height in meters squared and assigned to two categories: 1, overweight or obese (BMI ≥ 24); 0, otherwise (BMI < 24).

BP was measured following the 2018 Chinese guidelines for the management of hypertension: normal blood pressure (SBP < 120 mmHg and DBP < 80 mmHg); normal high values (SBP = 120–139 mmHg and/or DBP = 80–89 mmHg); hypertension (SBP ≥ 140 mmHg and/or DBP ≥ 90 mmHg) [28]. In brief, BP was measured twice or more by using a Mercury or automatic sphygmomanometer in a sitting position after a minimum of 5 min rest after the anthropometric measurements [18]. If the two readings of SBP or DBP differed by 5 mmHg, then the readings were measured again, and the average of the three readings was recorded [28].

### 2.4. Statistical Analysis

Data were analysed using SPSS for Windows software package version 20 and AMOS 20.0 (SPSS Inc., Chicago, Illinois). Frequency and percentage were utilised to describe the sociodemographic variables. Binary logistic regression was employed to examine the relationship between physical activity, obesity, and hypertension. Age, gender, education level, occupation, marital status, smoking history, drinking history, and family history of hypertension were included within all regression models to account for any confounding effects of these factors. Significance was set at *p* < 0.05.

## 3. Result

The mean age of the study population was 40.7 years (SD = 12.2), 53.2% of which were women. Half of the participants were engaged in the first type of occupation, such as agriculture, production, business, and service personnel. In terms of education level, only 43.9% reached at least junior high school. Men accounted for 98.7% of those who smoke. Similarly, more men than women drank alcohol. Notably, 80.9% reported no family history of hypertension. Of the total sample, 11.3% reported engaging in low physical activity levels, 32.1% in moderate physical activity levels, and 56.5% in high physical activity levels. Among the participants, 40.4% were overweight or obese, and 31.9% were hypertensive (Table 1).

Chi-square analysis revealed a correlation between gender (*χ*^2^ = 9.639, *P*=0.002), age (*χ*^2^ = 21.085, *P* < 0.001), education (*χ*^2^ = 24.348, *P* < 0.001), and hypertension. Moreover, the analysis found a significant association between smoking behaviour and hypertension (*χ*^2^ = 5.863, *P*=0.015). Family history of hypertension was significantly associated with the prevalence of hypertension. Furthermore, the results showed a close relationship between obesity and hypertension (Table 2).

The results of multivariable analysis of the influence of physical activity on obesity and hypertension are summarised in Table 3. In model 1, after adjusting for basic demographic characteristics, such as gender, age, education, occupation type, marital status, and individual behaviour, the effect of moderate and high physical activity on obesity was statistically significant (OR = 0.564, 95% CI: 0.352–0.905; OR = 0.583, 95% CI: 0.375–0.907). Model 2 revealed that the effect of high physical activity level on hypertension was statistically significant (OR = 0.556, 95% CI: 0.384–0.888).

**Figure 1:**
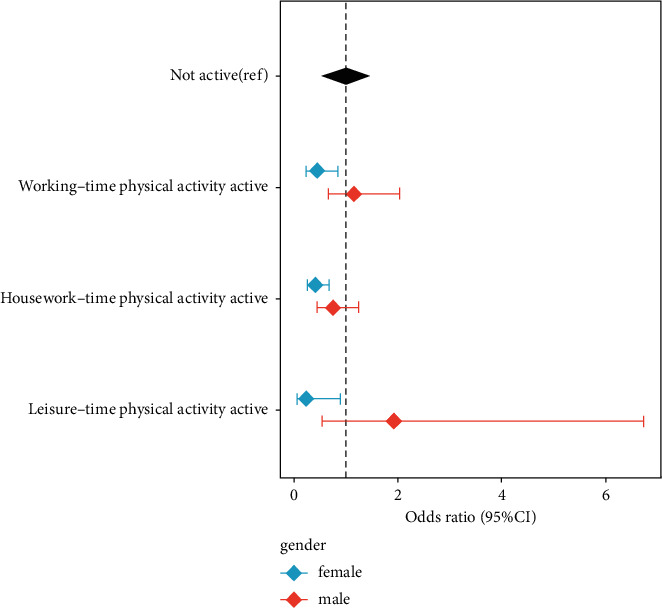
The impact of different types of physical activity on hypertension (adjusting for gender, age, occupational status, educational level, marital status, smoking, drinking, and family history of hypertension).

The effect of the type of physical activity on hypertension is depicted in Figure 1. Women were more sensitive than men to the effects of physical activity. After adjusting for the influence of confounding factors, physical activity at work, housework, and leisure were related to the prevalence of hypertension unlike physical inactivity. After adjusting for the effects of confounding factors, the results revealed a significant association between obesity and hypertension (OR = 0.700, 95% CI: 0.514–0.953) (Table 4).

After adjusting for confounding factors, such as gender, age, education level, marital status, smoking history, and drinking history, multivariable unconditional logistic regression analysis revealed that people with no hypertension family history with overweight/obesity were 58.5% less likely to have hypertension (OR = 0.415, 95% CI = 0.229–0.752) (Table 5).

## 4. Discussion

This study explored the relationship between physical activity and obesity and hypertension in the population in Pingshan District, Shenzhen City, China. Even after adjusting the sociodemographic characteristics of the respondents, a statistically significant difference was observed between the residents' physical activity levels and risk of obesity. Moderate and high physical activity levels were negatively correlated with the risk of obesity. The more active the residents were, the less likely they would be obese. Moreover, physical activity level was negatively correlated with the prevalence of hypertension, further verifying the protective effects of physical activity against hypertension [22, 29]. Physical activity is usually recommended as an important lifestyle modification that may help in preventing hypertension [20]. Furthermore, after adjusting for sociodemographic variables, women were found to be more sensitive than men to the effects of physical activity. In the family, aside from engaging in work and leisure, women generally do the housework. Household activities may increase overall physical activity because they require physical exertion, such as cooking, shopping, and gardening [30]. In addition, as long as a certain exercise intensity is reached, whether it is aerobic or resistance training, it will cause a significant increase in the content of *β*-endorphin in the female central nervous system, promote adaptive changes in the hypothalamic-pituitary-ovarian axis, and promote oestrogen in the body secretion [31]. Oestrogen is an important hormone involved in the metabolism of substances and energy in the body. Studies have found that the reduction of female oestrogen is closely related to the occurrence of abdominal obesity, insulin resistance, and related diseases [32]. However, moderate/vigorous physical activity cannot completely offset the increased risk of hypertension associated with overweight and obesity [33]. In general, the prevalence of hypertension in Pingshan District was 31.9%, which is higher than that in Shenzhen City (19.04%) and in the entire country (25.2%) [3, 34]. Pingshan District is in the early stages of urbanisation, with factory manufacturing as the primary industry in the area. Since this is a key residential area for migrant workers, the age structure is skewed towards younger adults. However, they are less educated, lack health awareness with insufficient investment in health, and engage in unhealthy lifestyle [35]. These factors may lead to higher obesity and hypertension rates in Pingshan District than those in other regions.

These findings were similar to those reported in Nigeria [36]. Studies have found that overweight and obese people are less likely to suffer from hypertension. This does not mean that obesity is a protective factor, because this is a cross-sectional study that cannot confirm its causality. It can only be said that overweight and obese people should strive to become physically active because physical activity will help in achieving physical fitness and ideal weight as well as reduce the risk of hypertension due to obesity, or obese people should pay more attention to their health [37]. Moon et al. [38] suggested that individuals with less physical activity and more sedentary behaviour may be more susceptible to genetic effects on adiposity. From another perspective, individuals with a greater genetic predisposition to obesity are more susceptible to the beneficial effects of physical activity and the deleterious effects of sedentary behaviour on adiposity [38]. Previous studies proposed that redox imbalance might be associated with the pathogenesis of hypertension, although it may not be the only cause of BP elevation [39–41]. Interestingly, physical activity has been suggested to be helpful in reducing the risk of hypertension by improving the redox state, particularly in the vascular wall [42, 43]. Therefore, aside from traditional pharmacological treatment modalities, physical exercise may be potentially important for preventing or treating hypertension or hypertension-related diseases [44]. These findings were similar to the results of the present study. People with more physical activity are less likely to be obese and develop hypertension. However, some studies believe that moderate exercise will enhance the body's immune function, while prolonged excessive exercise will cause the body to produce an “empty window period”, in which Th1 and Th2 are unbalanced, the number of NK cells decreases, and the CD4 + /CD8 + ratio decreases. The function of lymphocytes is inhibited. Especially for people who sit for a long time, strenuous exercise for a long time is not a suitable exercise intensity, which can easily lead to suppression of immune function. The exercise process needs to be carried out step by step [45, 46].

The present study also proved that people with a family history of hypertension are more likely to develop hypertension. Other studies obtained similar results [47]. Although family history is uncontrollable, the risk of being overweight or developing obesity can be reduced within the normal range through exercise and healthy diet. Therefore, people in high-risk groups should exercise and consume a reasonably healthy diet to reduce the risk of hypertension.

Notably, the residents who were overweight or obese were found to be less likely to develop high BP than those who had normal weight. Several reasons may possibly explain this result. Previous studies found that family history of hypertension and being overweight/obesity have an interactive effect on hypertension [47]. Among the overweight/obese people in the present study, 83.0% had no family history of hypertension, and only 17.0% had a family history of hypertension. In addition, people with no hypertension family history with overweight/obesity were 58.5% less likely to have hypertension. Furthermore, the question of whether obesity is a mediator between physical activity and hypertension was analysed further. However, hypertension is affected by many factors and is more sensitive to physical activity and family history of hypertension. Obesity is a complication or a health outcome of numerous diseases, and it may exist as an interfering factor between hypertension and physical activity. The present study did not gather information on the study population's drug usage/medications, so it is possible that hypertensive participants took antihypertensive drugs prior to BP measurements. In addition, participants may change their modern dietary patterns based on roasted/smoked foods, instant foods, and fermented foods to prevent and control the development of hypertension after illness, but the study did not collect this information. These possibilities will affect the results [48]. Therefore, this study was not able to establish the relationship between obesity as a mediator affecting physical activity and hypertension. This relationship can be investigated in a follow-up study. Determining this relationship may elucidate the mechanism by which physical activity influences obesity and hypertension.

This study has several strengths and weaknesses. Given that the district is still in its early stages of urbanisation, the government should plan this area in such a way that is conducive to physical activity. This study provides additional evidence that links physical activity, obesity, and hypertension and lays a foundation for future research. Moreover, this study offers a hypothesis on the relationship between physical activity, obesity, and hypertension that can be tested in the future. However, this study was only a small cross-sectional survey that excluded the determination of causality. Therefore, this study did not confirm the exact link between obesity and hypertension. In addition, the study did not gather other possible influencing factors, such as diet, medication, and salt intake, related to hypertension, which may have had an effect on the results. Finally, physical activity was obtained by self-reporting and questionnaires in the absence of professionals/objective instruments during measurements and recording. Hence, the data may have had inherent reporting bias. The objective and subjective physical activity levels of participants should be obtained with professional tools and questionnaires.

In the context of the “healthy city projects” of China, which attempt to shift the diagnosis and treatment of hypertension from “treatment-centred” to “health management-centred”, prevention should be advocated, and healthy lifestyles should be promoted [49]. In this context, the present findings are relevant in the development of infrastructures in Pingshan District in such a way that promotes physical activity among the residents. In addition, the results also provide a basis for the health departments in formulating strategies for preventing and controlling hypertension. Health managers should strengthen communication with residents, help them change their lifestyles, monitor their conditions, and provide necessary information and suggestions to improve residents' self-management skills and better comply with health management suggestions [50]. The age structure of Pingshan District is relatively young, and the typical “healthy immigration phenomenon” may conceal the potential of exposure to more relevant risk factors in the population. The incidence of chronic diseases is higher in younger populations, and thus relevant studies must be conducted in this area.

## Figures and Tables

**Table 1:** Individual characteristics of study participants, overall and by gender.

Variables	All		Male		Female		*P*
	*n*	%	*n*	%	*n*	%	
Age (years)							0.244
18–29	226	22.6	108	47.8	118	52.2	
30–44	374	37.4	187	50.0	187	50.0	
45–59	339	33.9	144	42.5	195	57.5	
≥60	62	6.2	29	46.8	33	53.2	

Educational level							<0.001
Primary school or below	218	21.8	74	33.9	144	66.1	
Junior high school	439	43.9	195	44.4	244	55.6	
High school and secondary school	239	23.9	140	58.6	99	41.4	
College degree and above	105	10.5	59	56.2	46	43.8	

Occupational status							0.181
First category	497	49.7	234	47.1	263	52.9	
Second category	174	17.4	71	40.8	103	59.2	
Third category	330	33.0	163	49.4	167	50.6	

Marital status							0.002
Married	851	85.0	415	48.8	436	51.2	
Unmarried	150	15.0	53	35.3	97	64.7	

Smoking							<0.001
Yes	224	22.4	221	98.7	3	1.3	
No	775	77.6	246	31.7	529	68.3	

Drinking							<0.001
Yes	150	15.1	133	88.7	17	11.3	
No	845	84.9	333	39.4	512	60.6	

Family history of hypertension							0.817
Yes	189	19.1	90	47.6	99	52.4	
No	799	80.9	373	46.7	426	53.3	

Physical activity level							0.005
Low level	111	11.3	56	50.5	55	49.5	
Moderate level	315	32.1	123	39.0	192	61.0	
High level	554	56.5	278	50.2	276	49.8	

Overweight or obesity							0.839
No	545	59.6	257	47.2	288	52.8	
Yes	370	40.4	177	47.8	193	52.2	

Hypertension							0.002
No	639	68.1	280	43.8	359	56.2	
Yes	300	31.9	164	54.7	136	45.3	

First category: agriculture, forestry, animal husbandry, fishery, production, transportation equipment operations, business, and service personnel. Second category: state agencies, institutions, civil servants, professional and technical personnel, and students. Third category: unemployment, housework, and retired persons.

**Table 2:** Univariate analysis of influencing factors of hypertension.

Variables	Hypertension		*χ* ^*2*^	*P*
	Yes (*n* (%))	No (*n* (%))		
Gender			9.639	0.002
Male	164 (54.7)	280 (43.8)		
Female	136 (45.3)	359 (56.2)		

Age (years)			21.085	<0.001
18–29	47 (15.7)	159 (24.9)		
30–44	99 (33.0)	248 (38.8)		
45–59	129 (43.0)	197 (30.8)		
≥60	25 (8.3)	35 (5.5)		

Educational level			24.348	<0.001
Primary school or below	89 (29.7)	120 (18.8)		
Junior high school	137 (45.7)	273 (42.7)		
High school and secondary school	56 (18.7)	165 (25.8)		
College degree and above	18 (6.0)	81 (12.7)		

Occupational status			5.384	0.068
First category	149 (49.7)	315 (49.3)		
Second category	63 (21.0)	100 (15.6)		
Third category	88 (29.3)	224 (35.1)		

Marital status			3.291	0.070
Married	245 (81.7)	551 (86.2)		
Unmarried	55 (18.3)	88 (13.8)		

Medical insurance			5.648	0.130
No	44 (14.7)	109 (17.1)		
Others	42 (14.0)	58 (9.1)		
Medical insurance for urban residents/new rural cooperative medical insurance	141 (47.0)	317 (49.6)		
Medical insurance for urban employees	73 (24.3)	155 (24.3)		

Smoking			5.863	0.015
Yes	82 (27.3)	129 (20.3)		
No	218 (72.7)	508 (79.7)		

Drinking			1.228	0.268
Yes	51 (17.0)	90 (14.2)		
No	249 (83.0)	543 (85.8)		

Family history of hypertension			16.401	<0.001
Yes	80 (27.1)	100 (15.8)		
No	215 (72.9)	532 (84.2)		

Overweight or obesity			7.878	0.005
Yes	97 (33.9)	268 (43.8)		
No	189 (66.1)	344 (56.2)		

Physical activity			4.099	0.129
Low level	42 (14.4)	65 (10.4)		
Moderate level	99 (33.9)	202 (32.2)		
High level	151 (51.7)	360 (57.4)		

**Table 3:** Multivariable analysis of the effects of physical activity on overweight/obesity and hypertension.

Variables	Overweight/obesity (model 1)		Hypertension (model 2)	
	OR	95% CI	OR	95% CI
Gender (ref = “male”)	0.928	0.656–1.311	0.525	0.360–0.764

Age (years) (ref = “18–29”)				
30–44	1.173	0.807–1.704	1.266	0.819–1.958
45–59	0.842	0.563–1.259	1.771	1.133–2.769
≥60	0.788	0.404–1.536	1.987	1.001–3.947

Educational level (ref = “primary school or below”)				
Junior high school	1.163	0.771–1.754	0.812	0.535–1.232
High school and secondary school	1.144	0.707–1.851	0.587	0.353–0.977
College degree and above	0.982	0.539–1.788	0.355	0.176–0.718

Occupational status (ref = “first category”)				
Second category	1.056	0.716–1.557	1.513	1.016–2.253
Third category	1.223	0.875–1.708	1.026	0.710–1.483
Marital status (ref = “married”)	1.150	0.751–1.761	1.315	0.839–2.060

Medical insurance (ref = “no”)				
Others	0.632	0.363–1.100	1.867	1.060–3.287
Medical insurance for urban residents/new rural cooperative medical insurance	0.918	0.623–1.353	1.043	0.680–1.600
Medical insurance for urban employees	0.642	0.410–1.003	1.245	0.768–2.020
Smoking (ref = “yes”)	1.072	0.714–1.609	0.975	0.640–1.485
Drinking (ref = “yes”)	1.335	0.875–2.037	0.902	0.584–1.394
Family history of hypertension (ref = “no”)	0.736	0.515–1.051	2.076	1.456–2.961

Physical activity level (ref = “low level”)				
Moderate level	0.564	0.352–0.905	0.697	0.425–1.144
High level	0.583	0.375–0.907	0.556	0.348–0.888

**Table 4:** Multivariable analysis of association between overweight/obesity and hypertension.

Variables	OR	95% CI
Gender (ref = “male”)	0.557	0.382–0.812

Age (years) (ref = “18–29”)		
30–44	1.114	0.725–1.711
45–59	1.553	0.997–2.418
≥60	1.394	0.700–2.776

Educational level (ref = “primary school or below”)		
Junior high school	0.732	0.481–1.113
High school and secondary school	0.564	0.338–0.942
College degree and above	0.375	0.187–0.750

Occupational status (ref = “first category”)		
Second category	1.529	1.024–2.285
Third category	1.033	0.713–1.497
Marital status (ref = “married”)	1.309	0.837–2.050

Medical insurance (ref = “no”)		
Others	1.847	1.032–3.306
Medical insurance for urban residents/new rural cooperative medical insurance	1.145	0.741–1.770
Medical insurance for urban employees	1.290	0.791–2.102
Smoking (ref = “yes”)	0.956	0.628–1.456
Drinking (ref = “yes”)	0.899	0.580–1.393
Family history of hypertension (ref = “no”)	2.059	1.439–2.946
Overweight or obesity (ref = “no”)	0.700	0.514–0.953

**Table 5:** The influence of family history of hypertension and overweight/obesity on hypertension.

Variables	Number of illnesses	OR	95% CI
Family history of hypertension and overweight/obesity	62	1.000	
Family history of hypertension and not overweight/obesity	114	1.183	0.615–2.276
No family history of hypertension and not overweight/obesity	425	0.644	0.366–1.133
No family history of hypertension and overweight/obesity	302	0.415	0.229–0.752

Adjusting for gender, age, occupational status, educational level, marital status, smoking, and drinking.

## Data Availability

The original data used to support the findings of this study are included within the article.
